# Effect of Negative Pressure Therapy on the Inflammatory Response of the Intestinal Microenvironment in a Porcine Septic Model

**DOI:** 10.1155/2015/419841

**Published:** 2015-07-30

**Authors:** Kenneth C. Norbury, Mary Pat Moyer

**Affiliations:** ^1^KCI, An Acelity Company, San Antonio, TX 78249, USA; ^2^INCELL Corporation LLC, San Antonio, TX 78249, USA

## Abstract

In a swine model of ischemia/reperfusion injury coupled with sepsis, we have previously shown attenuation of secondary organ injury and decreased mortality with negative pressure therapy (NPT). We hypothesized that NPT modulates the intestinal microenvironment by mediating the innate immune system. Sepsis was induced in 12 anesthetized female pigs. Group 1 (*n* = 6) was decompressed at 12 hrs after injury (*T*_12_) and treated with standard of care (SOC), and group 2 (*n* = 6) with NPT for up to *T*_48_. Immunoparalysis was evident as lymphocytopenia at *T*_24_ in both groups; however, survival was improved in the NPT group versus SOC (Odds ratio = 4.0). The SOC group showed significant reduction in lymphocyte numbers compared to NPT group by *T*_48_ (*p* < 0.05). The capacity of peritoneal fluid to stimulate a robust reactive oxygen species response *in vitro* was greater for the NPT group, peaking at *T*_24_ for both M1 (*p* = 0.0197) and M2 macrophages (*p* = 0.085). Plasma elicited little if any effect which was confirmed by microarray analysis. In this septic swine model NPT appeared to modulate the intestinal microenvironment, facilitating an early robust, yet transient, host defense mediated by M1 and M2 macrophages. NPT may help overcome immunoparalysis that occurs during inflammatory response to septic injury.

## 1. Introduction

Sepsis carries a high mortality rate and is the leading cause of death in critically ill patients [1–3]. Two independent septic disorders have been identified: “septic shock,” a highly lethal syndrome caused by cardiovascular collapse within 24–48 hours of onset, and “severe sepsis,” a more protracted condition associated with organ dysfunction that progresses more slowly over 7–14 days with a mortality rate of 30–70% [4]. The molecular biology of inflammation and sepsis is very complex and includes many mediators that qualitatively and quantitatively vary over time and physiological compartments [5]. There is overlap of certain stimuli, cytokines, and inflammatory factors with functions and markers that not only correlate with phenotypic and functional status but also reflect the timing and concentration of the stimuli and resulting host response. Upon recognition of a possible pathogen, resident macrophages within tissues initiate an early inflammation response that leads to recruitment of cells of the innate immune system, namely, neutrophils and monocytes [6], and to production of inflammatory cytokines, including interleukin (IL-1*β*), tumor necrosis factor (TNF), and high mobility group B protein-1 (HMGB1) [7]. The most common cause of lethal sepsis in human is bacterial contamination of the blood, causing an overwhelming “cytokine storm” that can rapidly result in multiple organ failure. Specific mediators and overt production of nitric oxide and reactive oxygen species (ROS) are key molecular mechanisms in the processes that can lead to tissue injury, including acute vascular and lung responses.

Serial analyses of monocyte phenotypes have been proposed for monitoring patients during acute phase and systemic disease and inflammatory responses [8]. The standard proinflammatory “M1” macrophage responses are induced by interferon gamma (IFN-*γ*) and lipopolysaccharide (LPS) and play a role in the immediate response to microbial pathogens that mediate the systemic inflammatory response syndrome (SIRS), whereas the “M2” alternate macrophage responses are seen in the compensatory anti-inflammatory response syndrome (CARS), parasitic infections, tumor growth promotion, and healing [9]. M1 and M2 macrophage activation states have been described in detail [10, 11]. The gut is one of the richest sources of macrophages in the body; the tissue microenvironment can markedly influence the phenotype of tissue-resident macrophages [10]. Reducing macrophage ROS levels in mitochondria results in defective bacterial killing [12]; hence, a robust production of ROS is key to bactericidal activity, especially under septic conditions. Therefore, a good understanding of the origins of these cells, as well as the timing and context of their recruitment, is essential not only to understand how tissues recover from exposure to proinflammatory stimuli but also to determine how these cells help to restore the status quo ante, which is important for diseases in which a loss of tissue homeostasis results in dysfunction of the tissue-resident macrophage populations [10]. Other factors or markers of sepsis progression and recovery, such as HMGB1 and Triggering Receptor Expressed on Myeloid Cells (TREM), are also important to matrix remodeling, tissue repair, and dampening of the immune response so that homeostatic levels are adequately restored.

The validity of cecal ligation and puncture as a rodent sepsis model is supported by studies where blocking TNF or LPS did not show any efficacy or prevent death [13]. More recently, a septic swine model was developed whereby intestinal ischemia/reperfusion and peritonitis resulted in massive systemic inflammation that led to abdominal compartment syndrome (ACS) [14]. ACS is known to significantly increase the morbidity and mortality of the trauma patient, including severe lung injury with pulmonary edema [15, 16] as part of the larger process of multiple organ dysfunction syndrome (MODS) [17]. Microcirculation in the gut is dramatically impaired [18, 19], which causes increased microvascular permeability, resulting in intestinal edema and peritoneal fluid (PF) formation [20]. The damaged gut is a continual source of local inflammation that can further promulgate SIRS leading to MODS and increased mortality [21–25]. The results of animal models of hemorrhagic shock, ACS, and sepsis are similar to those observed in human clinical trials and mimic polymicrobial infection, including the close resemblance of macrophage phenotypes [26, 27], making models useful for complex immunopathology studies of the nonlinear path of progression from SIRS to CARS [28–31]. CARS is associated with lymphopenia caused by massive apoptosis of circulating lymphocytes [32, 33] and immunoparalysis [34, 35]. The production of IL-10, yet another hallmark of CARS, can lead to immune paralysis [36–40]. Indeed, Olszak et al. [41] have shown that IL-10 in the intestinal epithelium plays a critical role in the regulation of mucosal homeostasis, which has broad implications for diseases such as inflammatory bowel disease. Finally, there is also a specific and physiological connection between the nervous and innate immune systems mediated by stimulation of the vagus nerve that leads to inhibition of proinflammatory cytokine production during infection and tissue injury [4]. Lymphocyte apoptosis can cause activation of macrophages to release inflammatory cytokines such as HMGB1 [42] and further exacerbates inflammatory responses by preventing the anti-inflammatory potential of the vagus nerve and cholinergic regulatory lymphocytes [43]. Prevention of lymphocyte apoptosis with caspase inhibitors can allow lymphocytes to act rapidly to control infection and can prevent mortality in experimental sepsis [42, 44].

For wounds requiring an open abdomen (OA), temporary abdominal closure (TAC) techniques are used to postpone fascial closure until predisposing factors causing pathologic elevation of intra-abdominal pressure and consequent ACS are resolved. These techniques include the Bogota bag [45], absorbable mesh, and the Wittmann patch or Velcro burr. Vacuum pack dressings and foam-based negative pressure wound therapy (NPWT) dressings [46] are TACs that apply negative pressure to help remove fluid from the OA. NPWT (V.A.C. Therapy, KCI, an Acelity company, San Antonio, TX) with the reticulated open-cell foam V.A.C. Abdominal dressing has evolved into negative pressure therapy (NPT, ABThera Open Abdomen Negative Pressure Therapy; KCI, an Acelity company, San Antonio, TX). The mechanism of action of NPT is believed to be in part due to the reduction of wound inflammation by its removal of wound exudates, whose chronic presence within a wound is deemed contraindicated for rapid and successful healing [14, 47]. Kubiak et al. [14] used a clinically relevant septic swine model developed by Nieman et al. [48, 49] to demonstrate that NPT improved survival by reducing MODS and significantly controlling the local and systemic inflammatory responses. In addition to active, efficient removal of the harmful inflammatory fluid from the peritoneal cavity, there remains the possibility that NPT alters the microenvironment such that the cells of the innate immune system are programmed to elicit a robust antimicrobial defense that is essential to the mitigation of sepsis in the face of the immunoparalysis that inevitably ensues as the host tries to stem the tide of a rapidly progressing cytokine storm.

We hypothesized that NPT modulates the intestinal microenvironment to better control sepsis as evidenced by a robust host defense response following septic injury and that this effect was mediated by the innate immune system. The observed beneficial effects of NPT might result from a dynamic programming of the innate immune cells that are recruited to the site of injury in the gut. This study used a porcine sepsis model to compare NPT to a standard of care (SOC), Bogota bag technique that does not utilize negative pressure, evaluating their effect on the local (peritoneal fluid (PF)) and systemic (plasma) microenvironments in regulating the respective* in vitro* inflammatory responses of human surrogate innate immune cells as well as clinically applicable outcomes of organ dysfunction (e.g., pathophysiology of the lung).

## 2. Materials and Methods

### 2.1. Animal Model

This study was approved by the institutional review board of the research laboratory and was conducted in adherence to appropriate guidelines. Fasted female domestic pigs (27–37 kg) were anesthetized with ketamine/xylazine (IM) and administered pentobarbital/ketamine (IV infusion) to maintain a surgical plane of anesthesia for the duration of the experiment. Animals were subject to orotracheal intubation and connected to a Narkomed anesthesia unit (Draeger Medical, Inc., Telford, PA) with initial settings during the surgical preparation as follows: tidal volume (*V*_*t*_) of 10 mL/kg, respiratory rate (RR) of 15 breaths/min, titrated to maintain *P*_*a*_CO_2_ within the normal range (35–45 cm H_2_O), and FiO_2_ of 21%. Under sterile conditions, a femoral artery catheter was placed for blood chemistry and gas content measurements (Heska, Loveland, CO) and mean arterial pressure (MAP) monitoring. A venotomy was performed on an external jugular vein for placement of a dual lumen catheter, allowing anesthesia, fluids, and antibiotic administration. In the contralateral external jugular a Swan-Ganz catheter (7 French) was placed for measurement of pulmonary artery and wedge pressures, sampling of mixed venous blood gases, and cardiac output (American Edwards 9520A Cardiac Output Computer, Irvine, CA). A Foley catheter was inserted into the bladder for measurement of urine output and was connected to a pressure transducer leveled at midline to measure intra-abdominal pressure. Animals received prewarmed lactate Ringer's solution (LRS, 35–37°C, IV) and a thermal line warmer was used for IV infusions to maintain normal body temperature. Intravenous LRS with a drip sufficient to keep the line open was used to maintain adequate hydration determined by physiology parameters (i.e., urine output and MAP). Hydration was deemed inadequate if urine output decreased to less than 0.5 cc/kg/hr or if MAP decreased to less than 60 mmHg. If either of these events occurred, pigs received an additional fluid bolus of LRS (250 mL, IV, followed by a second 250 mL if needed, with a maximum volume of 500 mL per hour). Fluid infusion was minimally recorded hourly. If pulmonary wedge pressure increased to 18 mmHg during bolus fluid administration, fluid administration was reduced to maintenance rate (10 mL/hr) until pulmonary wedge pressure dropped to ≤14 mmHg. If MAP was still below 60 mmHg or urine output was <0.5 cc/kg/hr bolus fluid administration was restarted. Fluid management was used to help maintain MAP from going below 60 mmHg; no vasopressors were used. The health of the animal was monitored closely throughout the experiment by normal hemodynamic and lung function parameters following instrumentation and normal blood gases and chemistries.

### 2.2. Surgical Procedure and Treatments

A midline laparotomy was performed, and the superior mesenteric artery was isolated and clamped for 30 min to induce intestinal ischemia confirmed by the loss of the mesenteric pulse as well as the discoloration of the bowel. After 30 min, the clamp was removed, and reperfusion was confirmed by the reappearance of the mesenteric pulse and the return of normal color to the bowel. At this point an enterotomy of 2 cm was performed to harvest feces (0.5 mL/kg) in the cecum and combined with 2 mL/kg of the blood to create a fecal-blood clot that was placed in the lower part of the peritoneal cavity. The cecum was closed and returned to the abdominal cavity and a catheter was placed and brought out through the skin for collection of peritoneal PF. Collected PF was flash-frozen for later measurement of inflammatory mediators. The abdomen was then closed with sutures and the time recorded as *T*_0_ (i.e., 0 hrs after injury). For the first 12 hrs of the protocol, the animals were treated in an identical fashion. All animals received the antibiotics ampicillin (2 g, IV bolus) and metronidazole injection (500 mg, IV per packaging instructions) administered at *T*_6_, *T*_18_, *T*_30_, and *T*_42_. The entire abdomen was reopened at *T*_12_, and animals were randomly assigned to treatment groups as follows: Control group (SOC; *n* = 6) had a Bogota bag plastic dressing sutured to the skin or fascia of the anterior abdominal wall (negative pressure was not applied). Treatment group (NPT; *n* = 6) had the ABThera Open Abdomen Negative Pressure Therapy System (KCI, an Acelity company, San Antonio, TX) applied to the open wound as per the manufacturer's instructions and a negative pressure (−125 mmHg) was maintained continuously for the duration of the experiment (up to 48 hrs of treatment). Lung function was monitored during the course of each experiment by calculating *P*_*a*_O_2_/FiO_2_, where *P*_*a*_O_2_ represents the partial pressure of arterial oxygen obtained from blood gas analysis and FiO_2_ is the fraction of inspired oxygen (1.0 = 100%). The ADI system (ADInstruments PowerLab/8sp data acquisition system and LabChart software, Australia) was used to continuously record hemodynamic and pulmonary data.

### 2.3. Clinical Pathology

Blood samples were collected at periodic intervals from each animal and evaluated using an Olympus AU400 Chemistry Analyzer and the ADVIA 120 Hematology Analyzer. Whole blood plasma samples were collected at regular intervals into tubes containing sodium heparin and centrifuged at 3500 rpm and 15°C for 10 minutes. Peritoneal fluid (PF) was collected through the abdominal catheter at the same time intervals as for plasma collection, following IP injection of 40 mL of warm sterile saline, and then centrifuged at 3500 rpm and 15°C for 10 minutes. Following centrifugation, plasma and PF were aliquoted and stored in sterile polypropylene tubes and frozen at or below −70°C until tested.

### 2.4. Description of Cell Lines and Their Use

In order to assess and compare the* in vitro* effects of porcine PF and plasma from the NPT and SOC groups on innate immunity, human surrogate cell types were selected. To generate M1 and M2 macrophages* in vitro*, the commercially available (American Type Culture Collection (ATCC) Manassas, VA) human U937 cell line (ATCC# CRL-1593.2) was used. This line is well published, including recent* in vitro* studies [50–52] showing that U937 cells can be induced with phorbol esters or interleukin-4 (IL-4), respectively, to reproducibly differentiate into M1 and M2 macrophage lineages. Likewise, the commercially available human HL-60 cell line (ATCC number CCL-240) has been extensively studied as a valid* in vitro* model, where there is also a large body of supportive literature, including recent* in vitro* studies [53], showing that HL-60 cells (about 80%) can be induced to differentiate into neutrophil-like cells by culture in the presence of all-trans-retinoic acid, dimethyl sulfoxide (DMSO), or other inducers. In this study, plasma and PF collected from septic swine were added to induce and control U937 and HL-60 cells.

### 2.5. Culture, Subculture, and Cryopreservation

Control cells were cultured in complete RPMI growth medium (Cellgro, Manassas, VA) in standard culture conditions (37°C in a humidified atmosphere of 5% CO_2_ : 95% air) supplemented with 10% fetal bovine serum (FBS: Fetal Plus, Atlas, Fort Collins, CO), 1 mmol/L sodium pyruvate (Cellgro, Manassas, VA), 0.1 mmol/L MEM with nonessential amino acids (Cellgro, Manassas, VA), and 2.0 mmol/L GlutaMAX (Life Technologies, Grand Island, New York). Cells were subcultured at 1 : 5 to 1 : 10 ratios. Seeding density was 2.0 × 10^5^/mL U937 cells, with 100 *μ*L per well of 96-well plates for induction studies. Reference cell bank stocks were cryopreserved in EZ-CPZ (INCELL Corporation LLC, San Antonio, TX).

### 2.6. Induction of U937 Cells to M1 and M2 Macrophages or Neutrophil Differentiation

#### 2.6.1. M1 Type Macrophages

Induction was done by growing U937 cells in complete RPMI medium and then shifting cells seeded in 96-well plates (2.0 × 10^5^/mL; 0.1 mL/well) to medium supplemented with 10 nM phorbol 12-myristate 13-acetate (PMA).

#### 2.6.2. M2 Type Macrophages

Induction was done by growing U937 cells in complete RPMI medium and then shifting cells in 96-well plates (2.0 × 10^5^/mL; 0.1 mL per well) to serum-free RPMI supplemented with 10 ng/mL recombinant human IL-4 (PeproTech, Rocky Hill, NJ).

#### 2.6.3. Neutrophils

Induction was done by growing HL60 cells in complete RPMI medium and then shifting cells seeded in 96-well plates (2.0 × 10^5^/mL; 0.1 mL/well) to medium supplemented with 1.2% v/v DMSO (Sigma, St. Louis, MO).

### 2.7. Postinduction Treatment Protocols

Cells were induced to differentiate as appropriate for the cell type for 4 days; no characterization using specific cell marker analysis was performed. Then the various source plasma and wound fluid specimens were added at a 1 : 20 dilution (5 *μ*L/100 *μ*L). After 24 hrs incubation, the culture supernatants were collected and stored in labeled plates at −80°C for assessment of ROS and a set of selected cytokines and biomarkers analyzed by immunoassays as described below. The cells were also lysed with RIPA protein extraction buffer (Life Technologies, Grand Island, New York) and stored at −80°C for later protein analysis studies.

### 2.8. ROS Assay

ROS was analyzed using the OxiSelect ROS Assay Kit (Cell Biolabs, San Diego, CA) following the manufacturer's instructions. In brief, target cells were induced for 4 days and then preloaded with the DCFH-DA dye in RPMI media without serum. After 24 hrs preloading, cells were rinsed twice to remove excess DCFH-DA dye. Serum-free RPMI medium was added (90 *μ*L/well). Multichannel pipetters were used to transfer 10 *μ*L into single wells of the assay plates containing the induced cells. After a 1 hr treatment, the reactions were stopped with cell lysis buffer and a freshly prepared DCF standard curve was added to the plate. Cellular esterase bioconversion of DCFH-DA to DCFH in the preloaded cells was detected using a SpectraMax M2 (Molecular Devices, LLC, Sunnyvale, CA) plate reader that provided fluorescence readings at 480 nm/530 nm, which were referenced to the standard curve. Data were captured on an Excel spreadsheet template and plotted as relative fluorescence values or “normalized” as indicated for Base Line (BL) values (plasma only) or time 0 as indicated.

### 2.9. Protein Microarray

To determine possible inflammatory mediators and available tools, we extensively reviewed the literature and made choices based on the following factors: (a) prior validation of the monocyte-like and neutrophil-like cell line models by several investigators; (b) commercial availability of cells and reagents and their established use for this type of research; and (c) the desire to develop human-based cell test systems that will provide robust, reproducible readouts to better allow interstudy comparisons and statistical analyses. An ELISA based multiplex array (Quansys Biosciences, Logan, UT) was used for the proteomic analysis in 96-well plates. The samples and a standard curve were applied to the plate and incubated. The plate was then washed and an HRP-conjugated secondary antibody mix used for the detection of the individual proteins. Chemiluminescent substrate was then added to the plate and the plate imaged and analyzed with a Q-View Imager and software (Quansys Biosciences, Logan, UT). The BCA Protein Assay was used to measure the amount of total protein in all samples [54]. The difference between NPT (T) and SOC (C) was calculated by determining the percent (%) change of C and T at 3 and 6 hrs of treatment from the start of treatment (*T*_0_) and subtracting C% change from T% change to get the T%-C% difference.

### 2.10. Euthanasia

Prior to euthanasia or immediately before death, 250–350 U/kg heparin was administered IV, allowed to circulate, and then the animal was euthanatized via an overdose of pentobarbital IV.

### 2.11. Statistical Analyses

All statistical analyses were carried out using SAS (SAS Institute Inc., Cary, NC) software release 9.1., and a statistical difference of *p* < 0.05 was considered significant. A log-rank test was performed for comparing the survival data, while Fisher's Exact test was performed for comparing mortality proportion of the study. Multiple measurements were taken for some parameters (e.g., ROS, physiology data). For animals that failed to survive the full 48 hrs of treatment, a least squares regression model was used to calculate the intra-animal predicted value. All recorded data with time points from initiation of treatment and later were used for data imputation. The primary analysis of repeated measures (RM) model for all time points during treatment was based on the individual data points at every 4 hrs of the imputed data. A 2-factor (treatment group and time {from the first time point after baseline to *T*_48_}) repeated measures analysis of covariance was performed using the baseline (*T*_12_, which represents the start of treatment) as the covariate based on predicted-imputed data. Significant results from the RM model were further examined at each time point using a Bonferroni correction to adjust for multiplicity.

## 3. Results

### 3.1. Mortality

**Figure 1:**
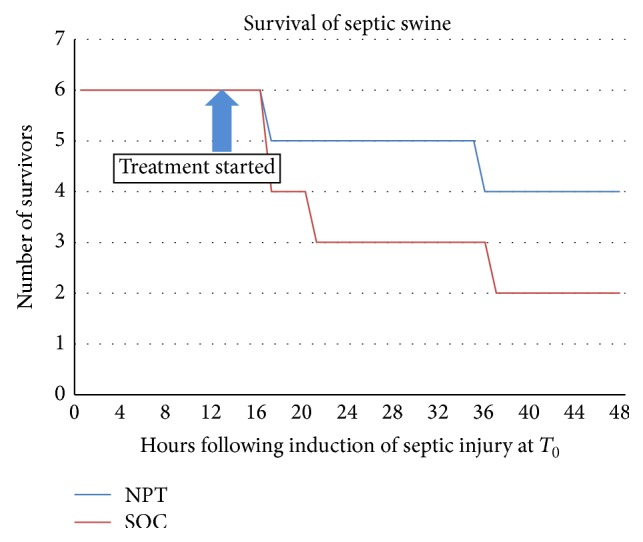
Survival rate of septic swine. The number of septic swine that survived the experimental period was determined for each treatment group. The mortality in the SOC group (4/6) was twice that of the NPT group (2/6). The Odds ratio was 4.0, indicating that survival was 4 times higher in the NPT group than in the SOC group.

The number of septic swine that survived the 48 hrs experimental period was determined for each treatment group (Figure 1). The mortality in the SOC group (4/6) was twice that of the NPT group (2/6). The average time of death was 40.8 ± 12.6 hours for the NPT-treated animals and 31.0 ± 15.0 hours for the SOC-treated animals. The *p* value for the log-rank test of survival was 0.253, and the *p* value for Fisher's Exact test of mortality proportion was 0.5671. However, the Odds ratio occurring in the NPT group compared to the SOC group was 4.0, indicating that survival was 4 times higher in the NPT group than in the SOC group. The Exact 95% Confidence Interval for Odds ratio is (0.23, 78.43).

### 3.2. MODS

**Figure 2:**
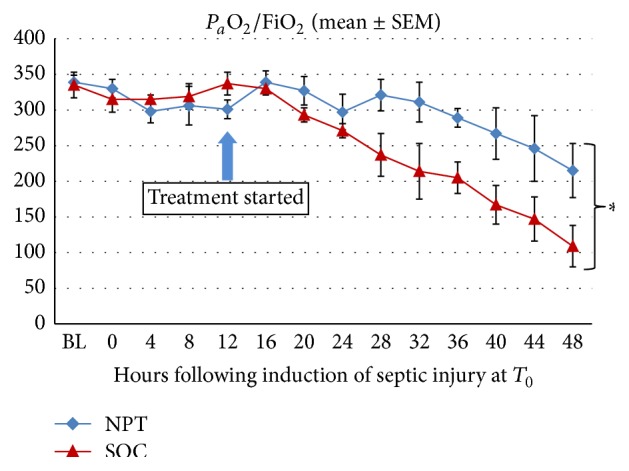
Lung function in septic swine. As a measure of MODS, lung function was determined by calculating the partial pressure of arterial oxygen divided by the fraction of inspired oxygen (*P*_*a*_O_2_/FiO_2_) at various intervals following the induction of septic injury. Lung function was severely compromised in the SOC group at 24 hrs following induction of septic injury as seen by a continual drop in *P*_*a*_O_2_/FiO_2_ from baseline (BL). However, NPT initiated at 12 hrs after injury not only appeared to reduce the severity of lung injury as seen by a delayed drop in *P*_*a*_O_2_/FiO_2_ but also resulted in significantly better lung function over approximately the last 20 hrs of the experimental period compared to the SOC group (^*∗*^ *p* = 0.0015).

As a measure of MODS, lung function was determined by calculating *P*_*a*_O_2_/FiO_2_ at various intervals following the induction of septic injury (Figure 2). Lung function was severely compromised in the SOC group after 24 hrs following induction of septic injury as seen by a continual drop in *P*_*a*_O_2_/FiO_2_. However, NPT initiated at 12 hrs after injury not only appeared to reduce the severity of lung injury as seen by a delayed drop in *P*_*a*_O_2_/FiO_2_ but also, compared to the SOC group, resulted in significantly better lung function over the last 20 hrs of the experimental period (*p* = 0.0015).

### 3.3. Hematology

**Figure 3:**
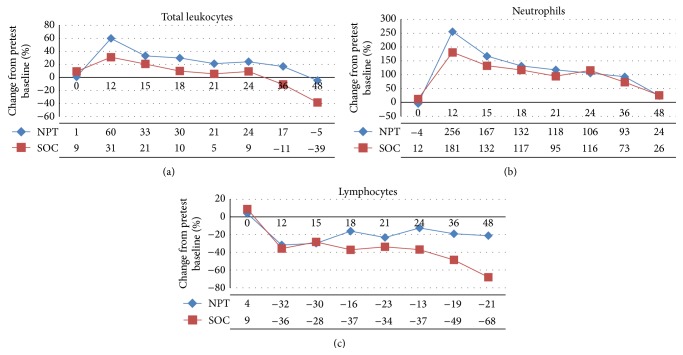
Hematology. The percent change (in the number of total circulating leukocytes, Panel (a)), neutrophils (Panel (b)), and lymphocytes (Panel (c)) in surviving animals over the duration of the experiment showed leukocytosis consisting predominantly of neutrophils that peaked at 12 hrs after injury and was observed during most of the experimental period in both treatment groups. Lymphopenia was observed beginning as early as 12 hrs after injury in both groups but progressed steadily in the SOC group, which was significantly greater than in the NPT group (^*∗*^ *p* < 0.05).

Leukocytosis, a reflection of the initial inflammatory response primarily mediated by neutrophils, was evident in both groups at 12 hrs after induction of injury, followed by a steady decrease coincident with treatment with SOC (Figures 3(a) and 3(b)). No difference between treatment groups was readily apparent. Immunoparalysis was observed as a decrease in the numbers of circulating lymphocytes (lymphopenia) beginning at 12 hrs following induction of septic injury. The number of circulating lymphocytes remained steady until *T*_24_ in the NPT group, while the SOC group showed a gradual and significant decrease over the latter stages of the experimental period (*p* < 0.05; Figure 3(c)).

### 3.4. ROS Production by Innate Immune Cells

**Figure 4:**
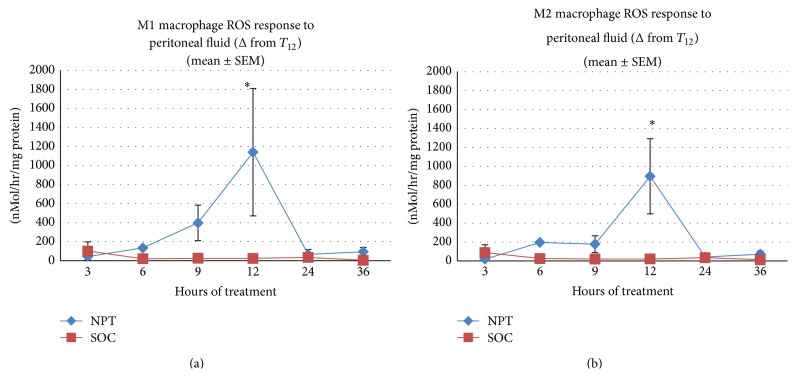
Production of ROS by M1 and M2 macrophages in response to peritoneal fluid (PF) from septic swine* in vitro*. The production of ROS by human macrophages was induced* in vitro* with PF taken from swine at various intervals following the induction of septic injury, and the results are expressed as the difference (Δ) from the initiation of treatment at the 12 hrs interval (*T*_12_), representing a 36 hrs period of treatment. (a) PF from NPT-treated septic swine induced a significant inflammatory response (mean change in nMol/hour/mg protein ± SEM from baseline) in M1 macrophages than PF from SOC-treated septic swine, peaking at 12 hrs after the initiation of treatment (^*∗*^ *p* = 0.019). (b) PF from NPT-treated septic swine induced a greater inflammatory response (mean change in nMol/hour/mg protein ± SEM from baseline) in M2 macrophages than PF from SOC-treated septic swine, peaking at 12 hrs after the initiation of treatment (^*∗*^ *p* = 0.085).

**Figure 5:**
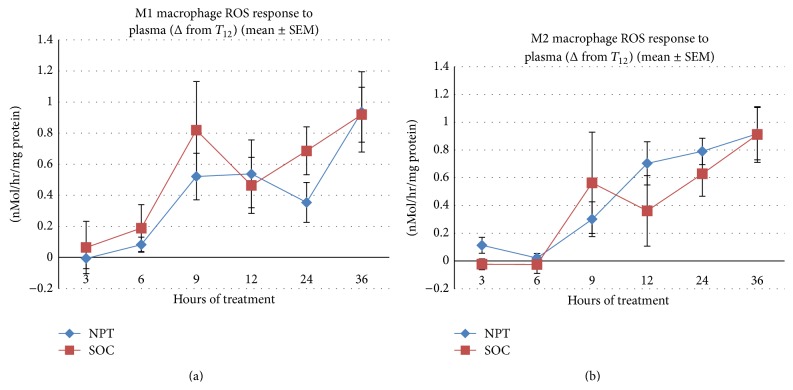
Production of ROS by M1 and M2 macrophages in response to plasma from septic swine* in vitro*. The production of ROS by human macrophages was induced* in vitro* with plasma taken from swine at various intervals following the induction of septic injury, and the results are expressed as the difference (Δ) from the initiation of treatment at the 12 hrs interval (*T*_12_), representing a 36 hrs period of treatment. There were no differences between treatment groups when M1 macrophages (a) or M2 macrophages (b) were incubated with plasma.

The production of ROS by human macrophages was induced* in vitro* with PF (Figure 4) or plasma (Figure 5) taken from swine at various intervals following the induction of septic injury, and the results are expressed as the difference (Δ) from the initiation of treatment at the 12 hrs interval (*T*_12_), which represents a 36 hrs period of treatment. PF from NPT-treated septic swine induced a significant inflammatory response (mean change in nMol/hour/mg protein ± SEM from baseline) in M1 macrophages in comparison to PF from SOC-treated septic swine, peaking at 12 hrs after the initiation of treatment (*p* = 0.019; Figure 4(a)). A similar trend was observed in M2 macrophages, again peaking at 12 hrs after the initiation of treatment (*p* = 0.085; Figure 4(b)). There were no differences between treatment groups when M1 macrophages or M2 macrophages were incubated with plasma (Figure 5), which is not unexpected at these early timepoints before SIRS is manifested. There were no significant treatment-related differences in ROS production by neutrophils (data not shown), which correlated with blood levels of neutrophils.

### 3.5. Inflammatory Protein Mediator Production by Innate Immune Cells

**Figure 6:**
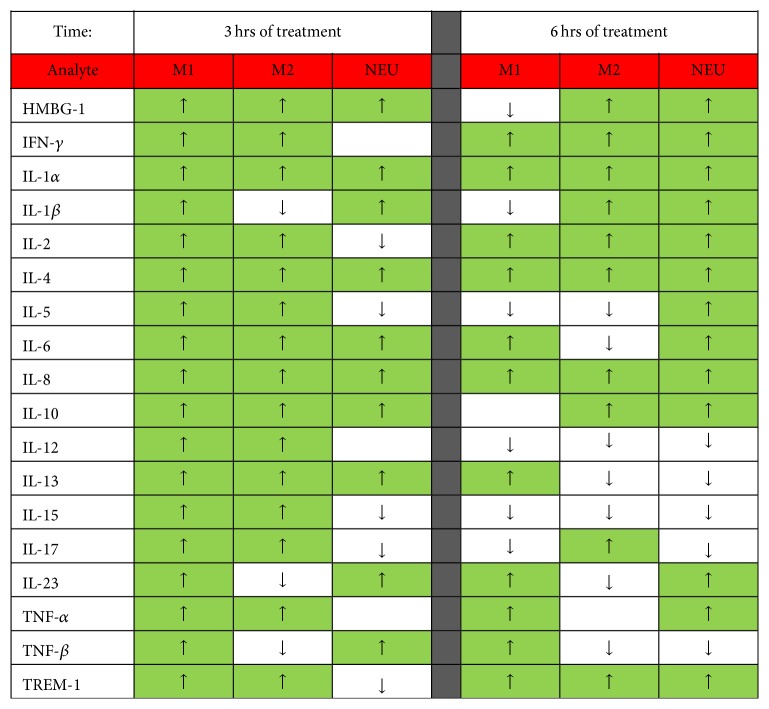
Inflammatory protein mediator heat map analysis of innate immune cells in response to peritoneal fluid (PF) from septic swine* in vitro*. The production of 18 different inflammatory proteins by human M1 and M2 macrophages and neutrophils (NEU) was induced* in vitro* with PF from swine taken at 3 hrs and 6 hrs intervals following initiation of treatment for septic injury induced at *T*_0_. Arrows indicate direction of response T-C (T = NPT; C = SOC). Green boxes highlight where T > C.

**Figure 7:**
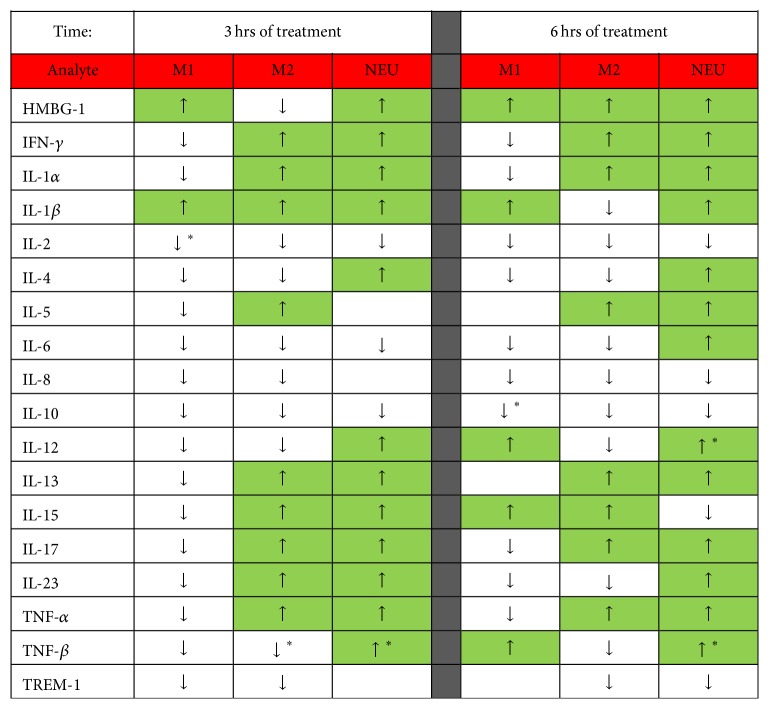
Inflammatory protein mediator heat map analysis of innate immune cells in response to plasma from septic swine* in vitro*. The production of 18 different inflammatory proteins by human M1 and M2 macrophages and neutrophils (NEU) was induced* in vitro* with plasma from swine taken at 3 hrs and 6 hrs intervals following initiation of treatment for septic injury induced at *T*_0_ (T = NPT; C = SOC). Arrows indicate direction of response T-C. Green boxes highlight where T > C.

The early robustness of the inflammatory response by human M1 and M2 macrophages and neutrophils was further demonstrated by protein microarray analysis of the effluent from cell cultures following induction* in vitro* by either PF (Figure 6) or plasma (Figure 7) from NPT-treated septic swine compared to SOC-treated septic swine. At 3 hrs after initiation of treatment (*T*_15_), the overall trend for PF from the NPT group revealed that, of 18 analytes examined, 18 and 15 analytes were induced to a greater extent by M1 macrophages and M2 macrophages, respectively, compared to PF from the SOC group (Figure 6). However, at 6 hrs after initiation of treatment only 11 and 10 of the analytes, respectively, were still elevated, showing a shift from a proinflammatory response towards a more anti-inflammatory response. Neutrophils were less responsive than macrophages at 3 hrs with 10/18 analytes elevated more by PF from NPT animals than SOC animals, while at 6 hrs 13/18 they were still elevated.

Plasma samples had an opposite effect upon macrophages. Plasma from the NPT group induced a lower inflammatory response than plasma from the SOC group at 3 hrs after initiation of treatment in 16/18 analytes produced by M1 macrophages (Figure 7). However, IL-10 and IL-2 produced by M1 macrophages were significantly elevated at 6 hrs and 3 hrs, respectively, while TNF-*β* from M2 macrophages was elevated at 3 hrs in the NPT group compared to the SOC group (*p* < 0.05). Neutrophils also showed a significantly increased production of IL-12 at 6 hrs and TNF-*β* at 3 and 6 hrs when incubated with plasma from the NPT group compared to the SOC group (*p* < 0.05).

## 4. Discussion

Using a swine model of ischemia/reperfusion injury coupled with severe, chronic sepsis due to the fact that the cecal enterotomy was left open when the abdomen was closed, we previously demonstrated increased proinflammatory response in the peritoneal cavity, secondary organ injury, and increased mortality, which were attenuated by NPT [14]. We hypothesized that NPT, compared to a TAC system that does not use negative pressure (SOC; Bogota bag) modulates the intestinal microenvironment in a dynamic, programmable manner via the innate immune system to better control sepsis.

For this study we modified the previous model [14] in order to achieve an even more clinically relevant model by closing the cecal enterotomy with sutures, thus creating an acute, rather than chronic, septic condition. Despite this modification, NPT was associated with increased survival, as reported by Kubiak et al. [14]. The improved survival due to NPT versus SOC in the septic swine model was directly associated with a reduction in MODS, as demonstrated by improved lung function (higher *P*_*a*_O_2_/FiO_2_ ratio) in the NPT group compared to the SOC group. No other physiology, clinical pathology, tissue edema, or histopathology parameters were significantly different between the 2 treatment groups, suggesting that septic injury was, as expected, not as severe in this “closed cecum” model compared to the “open cecum” model used by Kubiak et al. [14]. The improvement in survival was recently also observed in a clinical study of patients after surgery or traumatic injury that required open abdomen management and where subsequent treatment with NPT lasted at least 48 hrs [55].

The septic swine model also displayed evidence of leukocytosis in the initial 12 hrs after injury, followed by immunoparalysis manifested as lymphopenia, both of which are hallmarks of septic and traumatic injury to the abdomen. Leukocytosis represents the initial inflammatory response, while immunoparalysis is a result of a compensatory anti-inflammatory response designed to protect against an uncontrolled hyperinflammatory response. Whereas the SOC group showed a further decrease in the number of circulating lymphocytes as the experiment progressed, the NPT group showed a significant recovery by the end of the experiment, suggesting that NPT mitigated the further effects of systemic inflammatory injury and appeared to eventually overcome the effects of immunoparalysis in this model.

The exact NPT mechanism resulting in the improved survival and lung function outcomes in this septic swine model still needs to be fully elucidated. We hypothesized that NPT modulates the local inflammatory response in the gut, resulting ultimately in attenuation of the inflammatory response and preventing the spread of inflammatory mediators to secondary organs, such as the lung, where they can cause injury. We believe that the effect of NPT on the inflammatory response is not only due to the active removal of deleterious inflammatory mediators from the peritoneal cavity before they enter the systemic circulation as suggested by Emr et al. [56] but also due to a dynamic alteration of the microenvironment that enables a more robust antimicrobial response by the innate, and possibly adaptive, immune cells. This study specifically focused on the innate immune response by macrophages and neutrophils. Pig macrophages closely resemble human in their set of macrophage-expressed and LPS-inducible genes [27, 57]. The findings of this study showed that NPT modulates the inflammatory response in the intestinal fluid microenvironment to facilitate a robust, yet transient, innate immune response that transpires relatively early following septic injury at a critical stage when immunoparalysis is beginning to occur. For the purposes of this study, the neutrophils and macrophages may be viewed as representing surrogates for the freshly recruited cells, produced by the bone marrow via hematopoiesis, due to a combination of stress, traumatic injury, and leukopenia, which arrive in the microenvironment of the peritoneal cavity, where they are thus exposed to the inflammatory milieu (PF) therein. The production of ROS by macrophages was demonstrably greater when exposed to PF from NPT-treated animals than when exposed to PF from animals that received SOC. Peak responses occurred after 12 hrs of treatment and, most importantly, declined thereafter, suggesting that the response was neither uncontrolled nor hyperinflammatory. The protein microarray analysis highlighted the robust nature of the response by macrophages compared to neutrophils responding to the PF stimuli, particularly at the earlier time point (at 3 hrs of treatment), perhaps due to the overarching function of macrophages in the overall regulation of the inflammatory and repair process. The response to PF from NPT-treated septic swine was greater than the response to PF from SOC-treated septic swine and peaked early with the effect being even greater at 3 hrs than at 6 hrs of treatment, again suggesting that the inflammatory response is finite and is controlled. The inability of plasma to elicit as robust an inflammatory response in macrophages may be due in part to the fact that the systemic inflammatory response has not progressed to the degree of the local intestinal microenvironment at these early time points or inflammatory mediators are perhaps neutralized by plasma protease inhibitors. Nevertheless, these results support a similar temporal progression in terms of the levels of inflammatory mediators seen in the PF and plasma observed in the previous swine study by Kubiak et al. [14].

In the context of the observed immunoparalysis, the findings of this pivotal study suggest that NPT may have a dynamic effect on programming or maintaining a critical anti-infective innate immune response in the face of very predominant CARS being mounted by the host. Since the failure of the local inflammatory response to contain an infection is a key feature of the progression to sepsis [58–60], NPT could be quite beneficial in critically ill subjects via two key mechanisms of action: one is a more efficient and rapid removal of deleterious inflammatory PF, and the other is the creation of an intestinal microenvironment that is better suited to direct the innate immune response towards a more effective and timely resolution of the septic insult. Additional studies will help to further elucidate the mechanism of action of NPT and to confirm such effects in clinical cases.
